# Genome-Wide Association Study of Dietary Pattern Scores

**DOI:** 10.3390/nu9070649

**Published:** 2017-06-23

**Authors:** Frédéric Guénard, Annie Bouchard-Mercier, Iwona Rudkowska, Simone Lemieux, Patrick Couture, Marie-Claude Vohl

**Affiliations:** 1 Institute of Nutrition and Functional Foods (INAF), School of Nutrition, Laval University, Québec, QC G1V 0A6, Canada; frederic.guenard@fsaa.ulaval.ca (F.G.); Annie.Bouchard-Mercier@fsaa.ulaval.ca (A.B.-M.); Simone.Lemieux@fsaa.ulaval.ca (S.L.); 2 Endocrinology and Nephrology Unit, Centre de recherche du CHU de Québec, Laval University, Québec, QC G1V 4G2, Canada; Iwona.Rudkowska@crchudequebec.ulaval.ca; 3 Institute of Nutrition and Functional Foods (INAF), Endocrinology and Nephrology Unit, Centre de recherche du CHU de Québec, Laval University, Québec, QC G1V 4G2, Canada; patrick.couture@crchudequebec.ulaval.ca

**Keywords:** association study, dietary patterns, food preferences, Prudent, Western

## Abstract

Dietary patterns, representing global food supplies rather than specific nutrients or food intakes, have been associated with cardiovascular disease (CVD) incidence and mortality. The contribution of genetic factors in the determination of food intakes, preferences and dietary patterns has been previously established. The current study aimed to identify novel genetic factors associated with reported dietary pattern scores. Reported dietary patterns scores were derived from reported dietary intakes for the preceding month and were obtained through a food frequency questionnaire and genome-wide association study (GWAS) conducted in a study sample of 141 individuals. Reported Prudent and Western dietary patterns demonstrated nominal associations (*p* < 1 × 10^−5^) with 78 and 27 single nucleotide polymorphisms (SNPs), respectively. Among these, SNPs annotated to genes previously associated with neurological disorders, CVD risk factors and obesity were identified. Further assessment of SNPs demonstrated an impact on gene expression levels in blood for SNPs located within/near *BCKDHB* (*p* = 0.02) and the hypothalamic glucosensor *PFKFB3* (*p* = 0.0004) genes, potentially mediated through an impact on the binding of transcription factors (TFs). Overrepresentations of glucose/energy homeostasis and hormone response TFs were also observed from SNP-surrounding sequences. Results from the current GWAS study suggest an interplay of genes involved in the metabolic response to dietary patterns on obesity, glucose metabolism and food-induced response in the brain in the adoption of dietary patterns.

## 1. Introduction

Millions of people in both developed and developing countries are affected by cardiovascular diseases (CVDs), one of the world’s leading causes of morbidity and mortality [1]. Obesity is known to increase the risk of CVD [2] and a combination of decreased levels of physical activity and an increase in adverse eating behaviors contributes to the obesity pandemic [3]. There is also clear evidence of individual variability in response, suggesting that genetic susceptibility may have an important contribution to individual risk [4].

Single nutrients or food components have been studied to understand their impact on the development of chronic diseases [5,6]. Accordingly, individual dietary components have been associated with increased or decreased risk of diseases without consideration of the cumulative or synergistic effects of the consumption of multiple nutrients within a diet, a concept extensively discussed [5,6,7]. An alternative method for estimating diet may be to measure global food supply, thus taking into account the potential synergistic effects of multiple components within the diet [5]. One of the methods used to regroup foods that are consumed together involves factor analysis. This ‘a posteriori’ hypothesis-free derivation method uses observed/reported dietary data in order to extract dietary patterns [8]. Despite an ongoing debate on the validity of memory-based dietary assessment methods (M-BMs; e.g., 24-h dietary recalls and food frequency questionnaires (FFQs)), especially concerning their use in the formulation of national dietary guidelines [9,10], dietary patterns have been demonstrated to be concurrently valid and reproducible in comparison to other M-BMs [11], and are associated with CVD mortality [12] and risk factors such as diabetes, blood pressure, obesity and dyslipidemia [13,14,15]. Multiple studies summarized in a meta-analysis and in systematic reviews [16,17] identified the Prudent dietary pattern as a protective factor for CVD and reported an opposite relationship for the Western dietary pattern. The Prudent dietary pattern is mostly characterized by the consumption of vegetables, fruits, whole-grain products, fish and non-hydrogenated fats, whereas the Western dietary pattern is characterized by higher intakes of red meats, processed meats, refined grains, French fries and sweets/desserts [14,18].

Genetic variations in several genes were associated with macronutrient intakes such as protein, fat and carbohydrate [19,20]. Expanding single nutrients or food components, food preferences and dietary patterns were shown to be influenced by genetic variations [19,20]. Greater desirability for “unhealthy” food items was associated with gene variation in the dopamine-related *COMT* gene [21] and the rare allele of the rs9939609 single nucleotide polymorphism (SNP) in the fat mass and obesity-associated (*FTO*) gene has been associated with food preference; carriers of the rare allele consumed more biscuits and pastry and less soft drinks compared with TT carriers [20].

In line with abovementioned association of Prudent and Western dietary patterns with CVD and CVD risk factors, potential contribution of genetic susceptibility to CVD risk, and taking into account the debate on the validity of M-BM [9,10] combined with the lack of error-free, practical, and affordable method to assess whole dietary pattern data [22,23,24], our group previously demonstrated that gene expression profiles differed in individuals with high vs. low scores for both Prudent and Western dietary patterns [25], and that expression profiles may potentially modulate the risk of chronic diseases including CVD [25]. The current study aimed to assess the association of SNPs with the reported Prudent and Western dietary patterns scores. We conducted unbiased genome-wide approach and identified reported dietary pattern-related genetic variations. Further assessment of SNPs from associations identified was carried out through gene expression level and *in silico* analyses, and suggested interplay of genes involved in the metabolic response to dietary patterns in the adoption of dietary patterns.

## 2. Materials and Methods

### 2.1. Subjects

One hundred and forty-one individuals were selected among the 210 participants who completed the Fatty Acid Sensor (FAS) study, primarily aiming to understand how genes and environment act together to define CVD risk profile [26]. Individuals recruited in the FAS study had to be non-smokers and be free of any thyroid or metabolic disorders requiring treatment such as diabetes, hypertension, severe dyslipidemia, and coronary heart disease. A concurrently validated FFQ was administered by a registered dietician before omega-3 fatty acid supplementation [27]. Dietary patterns were derived by factor analysis from dietary intakes reported in FFQ. Further details on FAS study participants and recruitment criteria were published elsewhere [26]. This trial was registered at clinicaltrials.gov as NCT01343342. The subset of 141 individuals was originally selected among the FAS study participants based on DNA material availability and response to an *n*-3 polyunsaturated fatty acid supplementation [28]. The experimental protocol was approved by the Ethics Committees of Laval University Hospital Research Center and Laval University. The study was conducted in accordance with the Declaration of Helsinki and all participants provided written informed consent before their inclusion.

### 2.2. Anthropometric Measurements and Biochemical Profiling

Body weight (kg), height (m) and waist circumference (cm) were measured according to standardized methods [29]. Resting blood pressure (mm Hg) was measured in triplicate after a 10-min rest in a sitting position, phases I and V of Korotkoff sounds being respectively used for systolic (SBP) and diastolic (DBP) blood pressures [30]. Blood samples were collected prior the supplementation period from an antecubital vein into Vacutainer tubes (Becton, Dickinson and Company, Franklin Lakes, NJ, USA) containing ethylenediaminetetraacetic acid after a 12-h overnight fast and 48-h alcohol abstinence. Blood buffy coat and plasma were separated by centrifugation. Plasma total cholesterol (total-C, mmol/L) and triglyceride (TG, mmol/L) concentrations were measured using enzymatic assays [31] on an Olympus AU400e analyzer (Olympus America Inc., Melville, NY, USA). The high-density lipoprotein cholesterol (HDL-C; mmol/L) fraction was obtained after precipitation of very low-density lipoprotein cholesterol and low-density lipoprotein cholesterol (LDL-C) particles. LDL-C (mmol/L) was calculated with the Friedewald formula [32]. Fasting insulinemia (pmol/L) was measured by radioimmunoassay with polyethylene glycol separation [33] and fasting glucose concentrations (mmol/L) were enzymatically measured [34].

### 2.3. Dietary Assessment and Food Pattern Derivation

Habitual dietary intake for the month preceding the study was determined by a 91-item FFQ including 27 items with 1 to 3 sub-questions [27] and specifically based on food habits of Quebecers. This FFQ was previously shown to be reproducible and concurrently valid based on comparisons with a 3-day dietary record [27]. Participants had to answer to each question during a face-to-face interview with a registered dietician and were asked to report how often they consumed each type of food: daily, weekly, monthly or none at all during the last month. Examples of portion size were provided to ensure that each participant estimated correctly the proportion eaten. Information was compiled and the Nutrition Data System for Research software version 4.03 with Nutrient Database v2011 (Nutrition Coordination Center, University of Minnesota, Minneapolis, MN, USA) was used to analyze FFQ data. This database includes more than 16,000 food items with complete nutritional values for 112 nutrients. Similar food items from the FFQ were grouped, as previously described [14], and based on similarity of nutrient profiles, culinary usage and groups used in other studies [8]. Twenty-seven food groups were then formed and used for factor analyses to generate reported dietary patterns. The FACTOR procedure from Statistical Analysis Software (SAS) was used to derive factors from all participants considering eigenvalue >1, values at Scree test and interpretability to determine the number of factors to retain. Briefly, two main reported dietary patterns were derived. These patterns were similar to Prudent and Western dietary patterns from the literature [18]. Each individual was given a score for both reported dietary patterns. The SCORE procedure of SAS was used to calculate scores from the sum of food groups multiplied by their respective factor loading. These scores reflect the degree of each participant´s reported dietary intake conformance to a dietary pattern. Further details on reported dietary assessment, food grouping, food pattern derivation and factor loadings were provided elsewhere [25].

### 2.4. Genome-Wide Genotyping and Quality Control

DNA was isolated from blood buffy coats using the GenElute™ Blood Genomic DNA kit (Sigma, St. Louis, MO, USA). Quantification and verification of DNA quality were conducted via NanoDrop spectrophotometer (Thermo Scientific, Wilmington, DE, USA) and PicoGreen DNA methods. Illumina HumanOmni-5-Quad BeadChip^®^ (Illumina Inc., San Diego, CA, USA) were used to genotype more than 4,300,000 SNPs at the genome-wide level in the 141 individuals. Samples were tested for call rate (>95%) and gender mismatch based on genotyping data. All 141 samples were used in further analysis. Genotyping arrays were processed at the McGill University/Génome Québec Innovation Center (Montreal, QC, Canada) according to manufacturer’s recommendations. SNP allele frequencies and tests for Hardy–Weinberg equilibrium (HWE) were performed using PLINK [35] (version 1.07). SNP quality control was conducted and SNPs failing one of the criteria were excluded from analyses. Specifically, SNPs with insufficient call rate (<95%) or genotype distribution deviating from Hardy–Weinberg equilibrium (*p* values < 1.87 × 10^−8^) were excluded. In addition, monomorphic (non-variable) SNPs or with a minor allele frequency (MAF) < 0.01 were removed from analyses. Thus, a total of 1,632,526 SNPs were excluded, leaving 2,668,805 SNPs for statistical analyses.

### 2.5. Gene Expression Analyses

Pre-supplementation gene expression data were retrieved from previously published data [36] for 30 of the 141 individuals. Briefly, gene expression profiling was performed on RNA extracted from peripheral blood mononuclear cells using the Illumina Human-6 v3 Expression BeadChip and carried out at the McGill University/Génome Québec Innovation Center (Montreal, QC, Canada). The microarray data re-analysis was performed using the FlexArray software [37] and the Lumi algorithm. Robust multiarray average background adjustment was applied followed by log2 variance stabilization and quartile normalization. Transcripts were considered as expressed if they were detected in 25% of the samples.

### 2.6. Functional Analyses

Potential impacts of reported dietary patterns associated-SNPs herein identified on amino acid (aa) sequence and at protein level were analyzed using Variant Effect Predictor (VEP) [38]. Potential impacts of these SNPs on transcription factor (TF) binding sites and prediction of TF binding affinities based on DNA sequences were conducted using TRAP [39] an online tool comparing SNP surrounding sequences with known TF recognition sequences. TRAP has the capacity to identify TF binding sites among a SNP-surrounding sequence and to estimate TF affinity to the common and rare alleles. It also offers the possibility to identify overrepresented/enriched TFs among a group of sequences submitted, thus highlighting potential disruption of global regulators of biological mechanisms and providing biological insights for the associations identified. Sequences overlapping SNPs of interest (30 bp upstream and downstream) were submitted for analysis as input sequences. The Transfac vertebrates 2010.1 database was used as TF matrix file and human promoter sequences were introduced as background model.

### 2.7. Statistical Analysis

Clinical data were expressed as mean ± standard deviation for the full cohort and according to sex. Differences in clinical data between men and women were tested using Student’s *t*-test for continuous variables and Chi-square test for categorical variables. The general linear model (type III sum of squares) with adjustments for the effects of age, sex and body mass index (BMI) was used to test the associations of SNPs with CVD risk factors (fasting plasma lipids, glucose, insulin, SBP and DBP) as well as the associations of prudent and Western reported dietary pattern scores with these CVD risk factors. Transformations were applied for TG (logarithmic transformation; log10) and insulin levels (negative inverse transformation; 1/(-1*X)) to meet the criteria for normality. Partial Pearson correlations were computed to assess the relation between reported dietary pattern scores and associated CVD risk factors. Associations between SNPs and scores for prudent and Western reported dietary patterns were tested under linear regression using PLINK including age, sex and BMI as covariates. Nominal genome-wide association threshold of *p* < 1.0 × 10^−5^ was used to identify SNPs associated to reported dietary patterns. This significance threshold was used in order to avoid discounting true positive association based on the fact that statistical tests in genome-wide association studies (GWASs) are not independent due to linkage disequilibrium (LD) between SNPs and therefore the traditional method to adjust significance thresholds for multiple testing overcorrects when used in GWASs [40,41]. To evaluate the contribution of SNPs to the variance of reported dietary pattern scores, stepwise regression analysis was conducted. Differences in gene expression levels between genotype groups for reported dietary pattern-associated SNPs were tested using analysis of variance (general linear model, type III sum of squares) with adjustments for the effects of age, sex and BMI. LD (r^2^) between SNPs demonstrating significant associations was calculated from our data and from the 1000 Genomes Project phase 1v3 data [42] using Haploview [43] and LD calculator (https://caprica.genetics.kcl.ac.uk/~ilori/ld_calculator.php), respectively. SAS software version 9.3 (SAS Institute Inc., Cary, NC, USA) was used to test for differences in clinical data, associations and correlation of reported dietary pattern scores with CVD risk factors, and differences in gene expression levels according to genotype groups.

## 3. Results

### 3.1. Subjects’ Description

The current study included 141 individuals from a previously described supplementation study aimed at assessing gene–environment interactions on CVD risk profile [26]. Individuals included here were overweight, middle-aged men and women (68 men and 73 women; Table 1). Men and women had similar BMI, while men had higher SBP (*p* < 0.0001) and lower HDL-C levels than women (*p* < 0.0001). Comparing reported dietary pattern scores derived from dietary intakes reported for the month preceding the study, women were characterized by higher Prudent and lower Western scores than men (*p* = 0.04 and 0.01, respectively). When categorizing individuals with high (>0) vs. low (<0) scores for both reported dietary patterns, men were more prone to showing a high score for Western reported dietary pattern (*p* = 0.0006) while no difference between sex was identified for the Prudent reported dietary pattern score (*p* = 0.11).

### 3.2. Dietary Scores and CVD Risk Factors

Reported dietary pattern, characterized by high intakes of vegetables, fruits, whole grain products, non-hydrogenated fats for the Prudent and by high intakes of refined grain products, desserts, sweets and processed meats for the Western were tested for associations with CVD risk factors. Although limited by our sample size, the respective SNP frequency and their potential effect size, assessment of associations of Prudent score with CVD risk factors using correlation analysis revealed that DBP (*r* = −0.259, *p* = 0.002) and fasting insulin levels (*r* = −0.282, *p* = 0.0008), both showed inverse correlation with the Prudent score following adjustments for age, sex and BMI.

### 3.3. Association between SNPs and Reported Dietary Patterns

**Figure 1:**
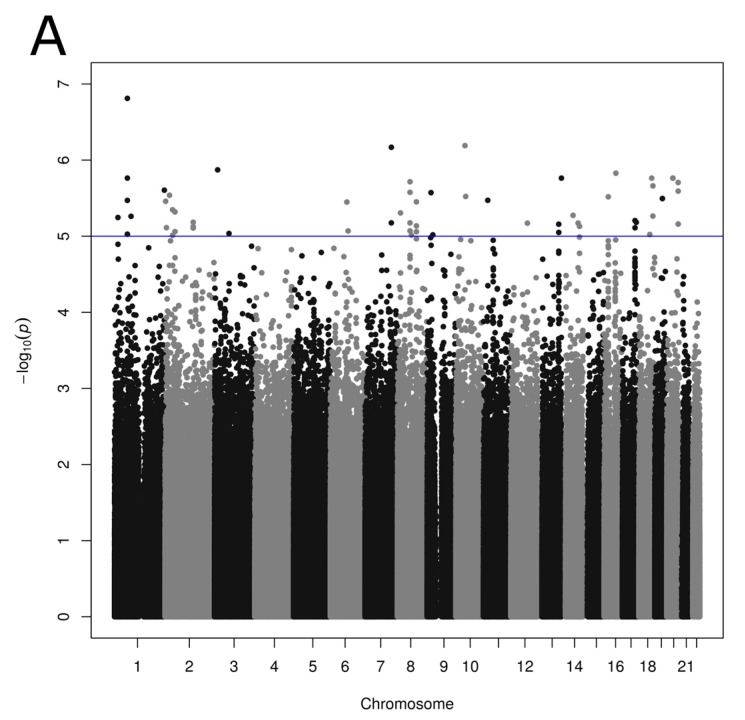

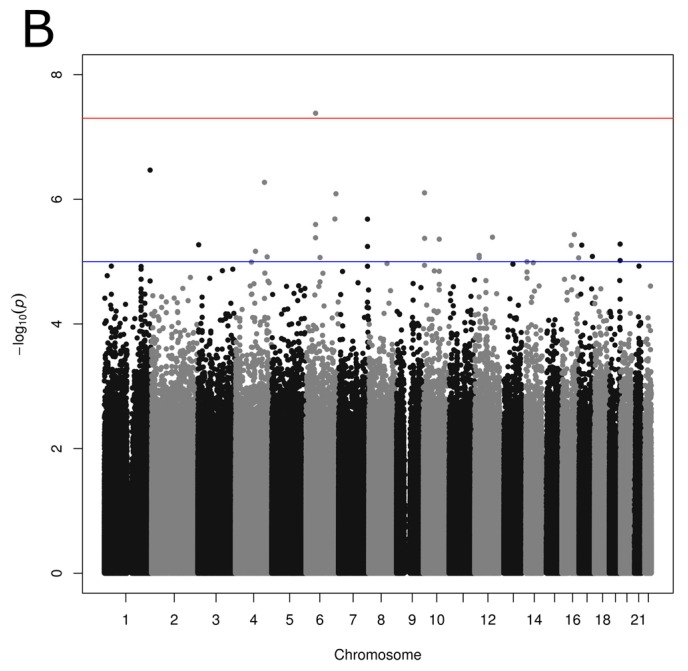
Manhattan plot showing *p* values obtained from genome-wide association studies between single nucleotide polymorphisms and reported dietary pattern scores: (**A**) Prudent; (**B**) Western reported dietary patterns. *p* values obtained using linear regression model with age, sex and BMI as covariates. Suggestive (*p* < 1.0 × 10^−5^) and conventional (*p* < 5.0 × 10^−8^) genome-wide association thresholds are represented by blue and red lines, respectively.

Associations were tested between 2,668,805 SNPs and each reported dietary pattern including age, sex and BMI as covariates. A total of 78 and 27 SNPs was associated with the Prudent and Western reported dietary pattern scores, respectively (*p* < 1 × 10^−5^; Figure 1, Tables S1 and S2). Associations identified were unique; none of the SNPs showed an association with both Prudent and Western scores. Low LD was generally observed in our study sample between Prudent-associated SNPs considering SNPs on the same chromosome, with few exceptions of large regions on chromosomes 2 (5 SNPs; 250 kb), 19 (3 SNPs; 118 kb) and 20 (12 SNPs; 476 kb) demonstrating strong LD (r^2^ ≥ 0.8). LD calculation from the 1000 Genomes Project data revealed moderate LD (r^2^ ≥ 0.6) between SNPs located within these regions. No such large region with strong LD was observed between SNPs associated with Western score with a mean LD of 0.23 in the present study sample.

SNPs associated with Prudent reported dietary pattern score were mainly located in gene regions, with 44 of the 78 Prudent score-associated-SNPs being located in gene regions. Most of these SNPs were intronic, while 5 were exonic, one was located in promoter and another in the 3’near gene region. Prudent-associated intergenic SNP rs13042507 is located near the *CTCFL* gene previously associated with type 2 diabetes (T2D) [44]. SNPs annotated to genes previously associated to obesity traits (*LINGO2* [45], *NELL1* [46]) and neurological disorders (schizophrenia (*ACSM1* [47], *KIF26B* [48], *NALCN* [49])), and alcohol and nicotine dependence (*LINGO* [50], *SH3BP5* [51]) were found among Prudent reported dietary pattern score associated-SNPs. SNPs associated to Western reported dietary pattern score were mostly intergenic; 19 of the 27 significant SNPs being intergenic while 7 were intronic and another was located in 3′ near gene region. SNPs from genes associated with alcohol dependence (*ESR1*) and obesity traits (*RGS7*, *NRG3* and *ESR1*) were observed among Western reported dietary pattern score-associated SNPs.

To get further insights on the contribution of SNPs in variability of reported dietary patterns, and to identify potential leading SNPs for regions demonstrating multiple significant associations, stepwise regression was performed from Prudent- and Western-associated SNPs. Among Prudent-associated SNPs, 14 SNPs contributed to explaining 76.2% of the Prudent reported dietary pattern score variability, while sex and BMI explained 2.0% and 1.0% of variability, respectively. From the 27 Western-associated SNPs, 9 explained 63.6% of variability in the Western reported dietary pattern score while confounding factors (age, sex, BMI) did not seem to contribute to variability. Potential leading SNPs revealed by stepwise regression analysis are highlighted in Tables S1 and S2.

### 3.4. Impact of SNPs on CVD Risk Factors

In order to test the potential implication of reported dietary pattern-associated SNPs in the associations between reported dietary patterns and CVD risk factors, we further tested reported dietary pattern-associated SNPs for associations with CVD risk factors. In line with associations identified here between Prudent reported dietary pattern scores and CVD risk factors (DBP and insulin), a total of three significant associations were identified with insulin levels (Table 2). Among these, the rs6499924 SNP located within *CNGB1*, showed the most significant association with insulin levels (*p* = 0.0005). Significant associations between SNPs located in the gluconeogenesis-regulating *PCK1* gene region and fasting glucose levels were also found although the Prudent reported dietary pattern score was not associated with fasting glucose levels in our previous analysis. Regarding Western-associated SNPs, five significant associations were identified between Western reported dietary pattern score-associated SNPs and total-C, including SNPs located within or near *RGS7*, *TET2*, *ARID1B* and *PFKFB3*.

### 3.5. Impact of SNPs on Gene Expression Level

**Figure 2:**
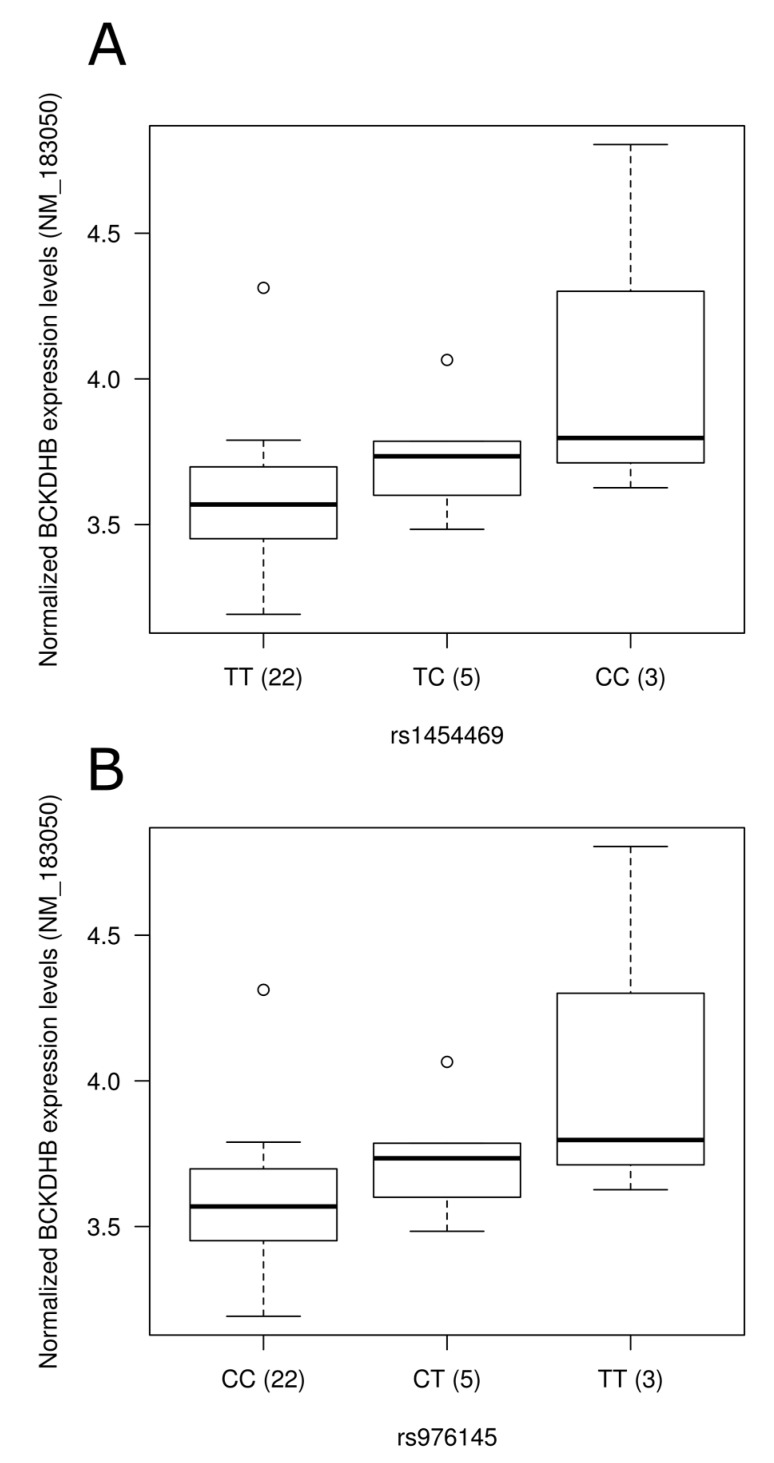

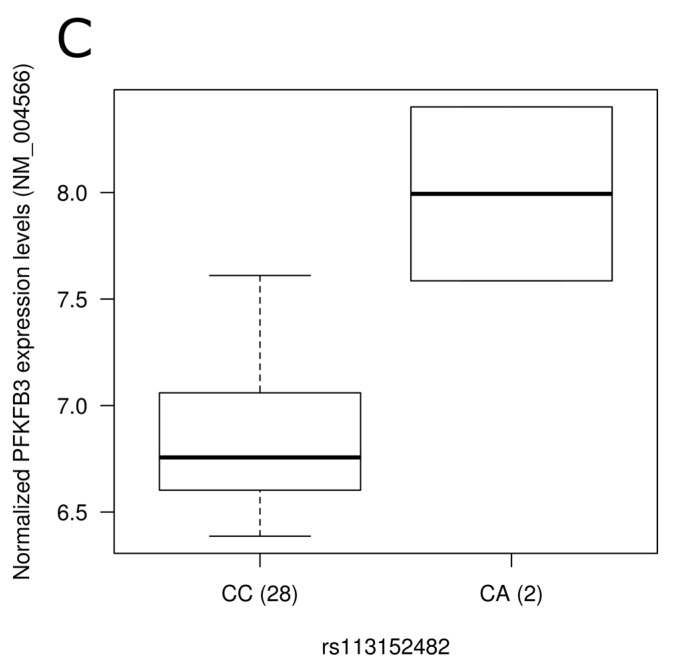
Representation of expression levels according to genotype groups for transcripts showing significant association with the presence of single nucleotide polymorphisms in blood. Expression levels of the *BCKDHB* gene (NM_183050) according to (**A**) rs1454469 and (**B**) rs976145 genotype groups; (**C**) Expression level of *PFKFB3* (NM_004566) according to rs113152482 genotype groups. Normalized expression levels are shown. The line in the middle of the rectangle represents the median while first and third quartiles are represented by box borders. Whiskers represent first and third quartiles ±1.5 interquartile ranges.

To assess the physiological impact of reported dietary pattern-associated SNPs and to provide potential molecular mechanisms underlying associations identified, we tested the association of SNPs with gene expression levels using gene expression data retrieved from a previous study [36] conducted on 30 individuals from our study sample (Table S3). Corresponding gene expression data were obtained for SNPs located in the gene region while expression levels of the nearest gene were retrieved for intergenic SNPs. Among genes annotated to diet associated-SNPs, 55 were found on gene expression array and 21 were detected in peripheral blood mononuclear cells. Despite few of the SNPs tested being associated with gene expression levels in this small study sample of 30 individuals, two intergenic SNPs associated with the Prudent reported dietary pattern (rs1454469, rs976145) were associated with expression levels (*p* = 0.02 for both) of *BCKDHB* (NM_183050). Rare allele carriers of these SNPs had higher expression levels (Figure 2A,B). These two SNPs demonstrated strong LD (r^2^ = 1.0) in our sample as well as in data from the 1000 Genomes Project. Testing Western reported dietary pattern-associated SNPs for association with expression levels, rs113152482 rare allele carriers showed higher *PFKFB3* (NM_004566) expression levels following adjustments for the effect of age, sex and BMI (*p* = 0.0004; Figure 2C). It is interesting to note that this SNP was highlighted by stepwise regression analysis as it contributed to 1.3% of the variability of the Western reported dietary pattern score.

### 3.6. Functional Analysis of SNPs

To provide further mechanistic insights for associations identified between SNPs, Prudent and Western reported dietary pattern scores, CVD risk factors and expression levels, we conducted TF analysis from SNP-surrounding sequences. Considering all Prudent-associated SNPs, FOXM1, glucocorticoid receptor (GR), CEBP and CEBPB were found among the most overrepresented TF binding sites. STAT family members and HMGA1 TFs were overrepresented from SNP-surrounding sequences for SNP associated with either Prudent or Western reported dietary patterns (Tables S4 and S5). IRF8 and PDX1 TFs were also overrepresented among surrounding sequences from SNPs associated with either the Prudent or the Western reported dietary pattern. Focusing on SNPs associated to CVD risk factors identified here, SNP rs6499924 associated with fasting insulin levels showed creation of potential GABP-alpha and ATF5 binding sites in the presence of the rare allele. Among glucose level-associated SNPs located in the *PCK1* gene region, rs6070157 resulted in aa change that was predicted to be tolerated or benign. For SNPs associated with gene expression, the rs976145 SNP associated to *BCKDHB* gene expression levels showed creation of HIF2A binding site while the presence of the rare allele of rs1454469 SNP, also located within the *BCKDHB* gene region, was predicted to disrupt IRX2 and IRX3 binding sites and to create a MEF2 binding site. Western reported dietary pattern-associated rs113152482 SNP, found to be associated with *PFKFB3* gene expression, showed the creation of a potential NFAT1 binding site.

## 4. Discussion

Using factor analysis from reported dietary intakes obtained from a concurrently validated FFQ [27], we first derived dietary patterns corresponding to Prudent and Western dietary patterns [14,18] previously reported to be associated respectively with protective and deleterious effects on CVD [16,17]. We thereafter tested associations between SNPs and reported dietary pattern scores using a nominal threshold of *p* < 1.0 × 10^−5^. This genome-wide association threshold was used to account the non-independency of statistical tests conducted [40,41] and combined with functional analyses to provide potential mechanistic insights for the associations identified. Although not reaching the conventional *p* < 5.0 × 10^−8^ GWAS significance threshold or Bonferroni corrected threshold, it provides clues for the discovery of biologically relevant associations. Identification of associations between reported dietary pattern-associated SNPs, CVD risk factors and gene expression levels argued for such biological importance of SNPs identified. Nonetheless, the most significant association observed here, between rs13212846 and the Western score (*p* = 4.16 × 10^−8^), reached a conventional *p* < 5.0 × 10^−8^ GWAS significance threshold. This SNP is located ~285 kb upstream the *DEFB112* gene encoding an antimicrobial and cytotoxic peptides made by neutrophils [52]. Another SNP located upstream of the *DEFB112* gene (~259 kb) was previously associated with BMI [53]. However, very low LD is observed between the BMI-associated rs17665162 SNP and the Western score-associated rs13212846 SNP herein identified. Globally, low LD observed between reported dietary pattern-associated SNPs and subsequent regression analysis demonstrated that a limited number of SNPs explains a large proportion of the variability in reported dietary pattern scores. These results suggest that: (1) some of the SNPs identified herein may act under an additive model; and (2) some other SNPs may act through common functional mechanisms with major SNPs potentially alleviating the impact of certain SNPs in common biological mechanism.

In line with the relationship between dietary patterns and CVD risk factors, the current study identified the rs13042507 SNP, near the *CTCFL* gene previously associated with T2D [44]. This SNP, herein associated with the Prudent reported dietary pattern, shows very low LD (0.008; 1000 Genomes Project data) with the rs328506 SNP associated with decreased risk of T2D [44], thus not allowing a potential biological link between Prudent dietary pattern and T2D-associated risk previously reported [54]. Nonetheless, association of reported dietary pattern-associated SNPs with CVD risk factors were also identified in the current study. Three SNPs (rs73180793, rs11552145, rs6070157) located within the gluconeogenesis-regulating *PCK1* gene region were found to be associated with fasting glucose levels. Although subjects recruited had to be non-diabetics, these associations are coherent with a potential association between *PCK1* SNPs and T2D [44], and between Prudent-like dietary patterns and decreased risk of T2D [54]. Testing Western reported dietary pattern -associated SNPs with CVD risk factors, the most significant association found involved the rs1348307 SNP located within the long intergenic non-protein coding RNA 706 (*LINC00706*) and fasting insulin levels (*p* = 0.0008). Although association between Western reported dietary pattern score and insulin levels was not observed in our cohort of overweight/obese men and women, such association of Western reported dietary pattern-associated SNPs with insulin level is coherent with a correlation of the Western score with insulin levels, as previously reported in men [55].

Mechanistic insights for the associations identified are provided herein through analysis of gene expression levels in blood and TF analysis. Increased expression levels of the *BCKDHB* gene (NM_183050) were observed in the presence of rare allele of Prudent reported dietary pattern-associated SNPs rs1454469 and rs976145, both SNPs demonstrating perfect LD in our study sample. Mutations in the *BCKDHB* gene are known to be responsible for the maple syrup urine disease (Online Mendelian Inheritance in Man #248600) characterized by mental and physical retardation, feeding problems, and a maple syrup odor of the urine. Specifically, the presence of SNP rs1454469 was predicted to create an MEF2 binding site. In *Caenorhabditis elegans* chemosensory neurons, MEF2 TF was recently found to be involved in sensory neuron–gut interaction, linking feeding state conditions to the regulation of chemoreceptor genes via insulin signaling [56]. An association between the Western diet associated-SNP rs113152482 and gene expression of *PFKFB3* in blood was also identified. *PFKFB3* encodes inducible 6-phosphofructo-2-kinase and is expressed in the brain [57]. It was shown to act as an essential glucosensor in hypothalamic neurons, linking glycolysis, AMP-activated protein kinase signaling and neuropeptide expression in mouse [58]. The rs113152482 SNP, highlighted by stepwise regression and explaining 1.3% of Western score variability, was predicted to disrupt the NFAT1 binding site. NFAT signaling plays critical roles in the development of multiple organ systems, including pancreas [59] and nervous system [60], and was reported to play a role in glucose homeostasis in pancreatic β-cells cellular models [61].

Having a global look at TF overrepresentation from surrounding sequences of reported dietary pattern-associated SNPs, overrepresentation of FOXM1 and GR TF were observed from Prudent reported dietary pattern-associated SNPs. FOXM1 is involved in cell proliferation, is necessary for the maintenance of adult beta-cell mass, beta-cell proliferation and glucose homeostasis, and was shown to be up-regulated in obesity [62]. Glucocorticoids (GCs) are known to mobilize the endocannabinoid system which is essential for negative feedback regulation of the hypothalamic–pituitary–adrenal axis [63]. In addition, a recent study using Cushing’s syndrome patients as a unique model of chronic GCs exposure demonstrated a negative correlation of urine cortisone with food-related choice thus implying a potential role of GR in food-choice behavior [64]. STAT family members and PDX1 TF were found to be overrepresented from SNP-surrounding sequences from both Prudent and Western reported dietary pattern-associated SNPs (Tables S5 and S6). STAT TFs were shown to be involved in energy homeostasis through an activation of the JAK-STAT pathway by leptin and their role in leptin-mediated satiety [65]. Specifically, STAT5 TF herein overrepresented is recruited by many hormones and cytokines that regulate food intake [66] whereas the PDX1 TF is involved in pancreatic development and glucose metabolism [67].

Results presented here tend to highlight a potential involvement of obesity-related and glucose metabolism genes in the adoption of dietary patterns concordant with a potential involvement of obesity genes in nutrient-specific food preference proposed following the analysis of obesity-associated loci revealed through genome-wide association study [19]. Notably, variants associated with body weight and BMI were previously reported to be associated with appetite, energy intake and eating behaviors [20,68], and several obesity genes were reported to be expressed in the hypothalamus, a center for energy balance and regulation of food intake. Specifically, interplay exists between food-induced brain responses and eating behaviour [69], and hypothalamus is a brain area specifically involved in food reward [70] thus potentially influencing food choice and the adoption of dietary patterns.

The current study used unbiased genome-wide approach to assess the genetics of the adoption of Prudent and Western reported dietary pattern scores. Results from the 91-items FFQ administered in the current study are based on reported data known to be biased by omissions, false memories, intentional misreporting and gross misestimation [9], and face-to-face interviews may have affected participants’ responses due to social desirability bias [71]. While these biases cannot be measured in the current study, the use of a population-specific FFQ [27] combined with an extensive database of food items with nutritional values available for 112 nutrients may partially alleviate the impact of self-reported nutritional assessment method on the derivation of reported dietary patterns. Despite subject to the imperfection of self-reported data and the ongoing debate on the validity of the memory-based dietary assessment methods [9,10], the concurrent validity and reproducibility of the FFQ used here were previously reported using a home- and self-completed 3-day food record [27], a dietary assessment method subject to recall bias, thus arguing for concurrent validity of the FFQ administered although validation was not performed in the current study and actual dietary intakes were not measured. Interactions between genetic and dietary factors as well as the impact of developmental processes on CVD risk factors were not analyzed, the main objective of the study being to identify associations between SNPs and reported dietary patterns to provide novel potential targets and biological mechanisms for CVD prevention. Since differences in reported dietary pattern scores between men and women have been identified herein from reported dietary intakes, sex has been included as a covariate in genome-wide analyses. However, analyses have not been conducted separately in men and women. BMI was also included as a covariate in our analysis, suggesting that association identified are BMI independent. However, we acknowledge that other CVD risk-associated confounding factors, e.g., developmental programming [72,73] and physical activity [74], were not taken into account for testing associations between reported dietary pattern-associated SNPs and CVD risk factors. Further generalization of conclusions at the population level merits further validation in general population, our cohort being composed of overweight individuals. An impact of SNPs on blood cell expression levels was observed here for a limited number SNPs. Nonetheless, we cannot rule out the possibility that they may exert their effect in other tissues.

Collectively, the association of SNPs with reported dietary pattern scores, CVD risk factors and expression levels argues for an impact of genetic variations on the determination of the adoption of Prudent and Western dietary patterns. Integration of association, expression and transcription factor data tends to reveal the involvement of obesity, glucose metabolism and neurological genes in the adoption of dietary patterns. As proposed herein, reported dietary pattern-associated SNPs may potentially act through an impact on glucose metabolism and food- and energy-sensing pathways.

## Figures and Tables

**Table 1:** Description of the genotyping cohort.

Characteristics	All	Men	Women
Number	141	68	73
Age (years)	31.6 ± 8.8	31.1 ± 8.0	32.0 ± 9.6
BMI (kg/m^2^)	28.4 ± 3.8	28.1 ± 3.7	28.7 ± 3.8
Waist girth (cm)	94.5 ± 11.0	96.3 ± 11.2	92.9 ± 10.7
Lipid profile			
Total-C (mmol/L)	4.90 ± 0.97	4.88 ± 1.03	4.92 ± 0.92
LDL-C (mmol/L)	2.88 ± 0.88	3.01 ± 0.95	2.75 ± 0.80
HDL-C (mmol/L)	1.42 ± 0.38 ^b^	1.25 ± 0.29	1.58 ± 0.39
TG (mmol/L)	1.32 ± 0.68	1.37 ± 0.72	1.27 ± 0.65
Total-C/HDL-C	3.66 ± 1.10 ^b^	4.10 ± 1.16	3.25 ± 0.88
Blood pressure (mm Hg)			
SBP	113.0 ± 12.3 ^b^	118.5 ± 12.9	107.8 ± 9.1
DBP	68.5 ± 8.4	68.7 ± 8.6	68.3 ± 8.3
Fasting glucose (mmol/L)	5.00 ± 0.46	5.06 ± 0.47	4.94 ± 0.44
Insulin (pmol/L)	93.2 ± 87.5	100.6 ± 119.2	86.4 ± 39.6
Self-reported diet scores			
Prudent	−0.022 ± 1.012 ^a^	−0.207 ± 1.041	0.150 ± 0.960
High/low scores (>0)	70/71	29/39	41/32
Western	−0.009 ± 0.980 ^a^	0.202 ± 1.087	−0.207 ± 0.829
High/low score (>0)	70/71^2^	44/24	26/47

Values presented (means ± standard deviation) are untransformed and unadjusted. Sex differences are identified. ^a^ *p* value < 0.05. ^b^ *p* value < 0.0001 Abbreviations: BMI, body mass index; Total-C, total cholesterol; LDL-C, low-density lipoprotein cholesterol; HDL-C, high-density lipoprotein cholesterol; TG, triglycerides; SBP, systolic blood pressure; DBP, diastolic blood pressure.

**Table 2:** Significant associations identified between reported dietary pattern-associated SNPs and cardiometabolic risk factors.

SNP ID ^a^	rs Number	Gene	Associated Pattern	Total-C	LDL-C	HDL-C	Total-C/HDL-C	SBP	Fasting Glucose	Insulin
kgp4289407	rs114123656	*LINC01246* ^b^	Prudent	---	---	---	---	---	---	0.02
kgp6444538	rs115510004	*LOC645949* ^b^	Prudent	---	---	0.008	---	---	---	---
rs10097298	rs10097298	*LOC100130298* ^b^	Prudent	---	---	---	---	---	0.03	---
kgp2826446	rs76838052	*C10orf142* ^b^	Prudent	---	0.02	---	---	---	---	---
kgp9480999	rs74842138	*GDF10* ^b^	Prudent	0.04	---	---	---	0.03	---	---
rs7144547	rs7144547	*STON2*	Prudent	---	---	0.004	0.03	---	---	---
rs163269	rs163269	*ACSM1*	Prudent	---	---	---	---	---	---	0.02
rs6499924	rs6499924	*CNGB1*	Prudent	---	---	---	---	0.04	---	0.0005
kgp5504930	rs13042507	*CTCFL* ^b^	Prudent	---	---	---	---	---	0.02	---
kgp6972810	rs73180793	*PCK1* ^b^	Prudent	---	---	0.02	---	---	0.02	---
kgp12008054	rs6070157	*PCK1*	Prudent	---	---	0.02	---	---	0.02	---
kgp10614850	rs11552145	*PCK1*	Prudent	---	---	0.04	---	---	0.01	---
kgp9374426	rs116812750	*RGS7*	Western	0.02	0.009	---	---	0.01	---	---
kgp8978882	rs112040989	*LOC101929468* ^b^	Western	0.03	---	---	---	0.02	---	---
kgp9399667	rs112764838	*TET2* ^b^	Western	0.03	---	---	---	0.02	---	---
kgp9469075	rs72736220	*LOC100996286* ^b^	Western	---	---	---	---	0.03	---	---
kgp29240591	rs148696004	*TLL1* ^b^	Western	---	---	---	---	---	0.01	---
kgp9282379	rs200247	*TFAP2D* ^b^	Western	---	---	---	---	0.04	---	---
kgp9033598	rs79041188	*ESR1*	Western	---	---	---	---	0.05	---	---
kgp26148321	rs141382233	*ARID1B* ^b^	Western	0.02	0.01	---	---	---	---	---
kgp4441528	rs2535974	*ACTR3B* ^b^	Western	---	---	---	---	0.008	---	---
kgp1054774	rs113152482	*PFKFB3*	Western	0.03	---	---	---	---	---	---
rs1348307	rs1348307	*LINC00706* ^b^	Western	---	---	---	---	---	0.006	0.0008
rs7911681	rs7911681	*NRG3*	Western	---	---	---	---	---	0.03	---
kgp6498073	rs112633616	*LOC101928441* ^b^	Western	---	0.03	---	---	---	---	---
kgp27660318	rs140957346	*EEA1*	Western	---	---	---	---	---	0.03	---
kgp25610618	rs140552175	*LOC101928880*	Western	---	0.05	---	---	0.02	0.002	---

^a^ SNP ID and annotated genes according to Illumina^®^ HumanOmni5-Quad BeadChip. ^b^ Nearest gene according to Illumina^®^ HumanOmni5-Quad BeadChip annotations. Significant *p* values are shown while non-significant ones are represented by dashes (---). No significant associations between SNPs and BMI, triglyceride levels or DBP were found. Abbreviations: SNP, single nucleotide polymorphism; Total-C, total cholesterol; HDL-C, high-density lipoprotein cholesterol; LDL-C, low-density lipoprotein cholesterol; SBP, systolic blood pressure; DBP, diastolic blood pressure.

## Supplementary Materials

The following are available online at www.mdpi.com/2072-6643/9/7/649/s1, Table S1: Associations identified between SNPs and Prudent dietary pattern, Table S2: Associations identified between SNPs and Western dietary pattern, Table S3: Description of gene expression cohort, Table S4: Transcription factors overrepresented in surrounding regions (60 bp) of Prudent dietary pattern-associated SNPs, Table S5: Transcription factors overrepresented in surrounding regions (60 bp) of Western dietary pattern-associated SNPs.
