# Supplementary material for: Genome-Wide Association Study of Dietary Pattern Scores
Source: Nutrients. 2017 Jun 23;9(7):649. doi: 10.3390/nu9070649 (PMC5537769; doi:10.3390/nu9070649)
Supplement: Supplementary file 1 [file nutrients-09-00649-s001.zip › nutrients-193524-supplementary.pdf]

Table S1. Associations identified between SNPs and Prudent dietary pattern.

SNP ID[a]	rs number	Position[b]	*P* value	Localization	Annotated gene	NearestGene
kgp1355428	rs76500500	chr1:63733119	$1.55x10^{-07}$	Intron	LINC00466	
kgp2826446	rs76838052	chr10:44797954	$6.45x10^{-07}$	Intergenic		C10orf142
**rs691184**	**rs691184**	chr7:129197408	$6.79x10^{-07}$	Intergenic		SMKR1
kgp6158523	rs139690209	chr3:15362622	$1.34x10^{-06}$	Intron	SH3BP5	
**rs6499924**	**rs6499924**	chr16:57970865	$1.49x10^{-06}$	Intron	CNGB1	
**rs12733446**	**rs12733446**	chr1:63666050	$1.72x10^{-06}$	Intron	LINC00466	
kgp1202278	rs17338417	chr13:115037377	$1.73x10^{-06}$	Intron	CDC16	
kgp4916880	rs116907056	chr18:63059975	$1.73x10^{-06}$	Intergenic		CDH7
kgp19320517	rs115384205	chr20:29860895	$1.73x10^{-06}$	Intergenic		DEFB115
kgp11433779	rs386813671; rs115591886	chr20:29893502	$1.73x10^{-06}$	Intron	DEFB116	
kgp10839569	rs114805574	chr20:29910596	$1.73x10^{-06}$	Intergenic		DEFB116
kgp4584282	rs113367131	chr20:29954283	$1.73x10^{-06}$	Promoter	DEFB118	
kgp4315795	rs17122694	chr20:29956736	$1.73x10^{-06}$	Intron	DEFB118	
kgp9682264	rs34247288	chr20:29960787	$1.73x10^{-06}$	Exon	DEFB118	
kgp2740564	rs115145768	chr20:29962345	$1.73x10^{-06}$	3' near gene region	DEFB118	
rs17090740	rs17090740	chr20:29995517	$1.73x10^{-06}$	Intron	DEFB121	
kgp10218471	rs114225688	chr20:30033885	$1.73x10^{-06}$	Intron	DEFB123	
kgp8087247	rs116990630	chr20:30253092	$1.73x10^{-06}$	Exon	BCL2L1	
kgp12006704	rs116949613	chr20:30286932	$1.73x10^{-06}$	3'UTR	BCL2L1	
kgp8018949	rs139737644	chr20:30336668	$1.73x10^{-06}$	Intron	TPX2	
rs6471915	rs6471915	chr8:61994781	$1.93x10^{-06}$	Intergenic		LOC100130298
**kgp10614850**	**rs11552145**	chr20:56138648	$1.97x10^{-06}$	Exon	PCK1	
kgp11174359	rs111297422	chr18:68974849	$2.18x10^{-06}$	Intergenic		LINC01541
**kgp10639373**	**rs10924198**	chr1:245600716	$2.48x10^{-06}$	Intron	KIF26B	
kgp5504930	rs13042507	chr20:56102821	$2.55x10^{-06}$	Intergenic		CTCFL
**rs10097298**	**rs10097298**	chr8:61992256	$2.65x10^{-06}$	Intergenic		LOC100130298
kgp10023042	rs113983136	chr9:18919204	$2.68x10^{-06}$	Intergenic		ADAMTSL
kgp3745359	rs111663987	chr9:18923128	$2.68x10^{-06}$	Intergenic		SAXO1
kgp6444538	rs115510004	chr2:21762805	$2.90x10^{-06}$	Intergenic		LOC645949
kgp9480999	rs74842138	chr10:48499927	$3.01x10^{-06}$	Intergenic		GDF10
rs163269	rs163269	chr16:20677170	$3.04x10^{-06}$	Intron	ACSM1	
kgp8761639	rs117875908	chr19:37549756	$3.20x10^{-06}$	Intergenic		ZNF420

kgp386428	rs148950464	chr19:37646755	3.20x10^{-06}	Intron	ZNF585A	
kgp3321820	rs117896655	chr19:37667428	3.20x10^{-06}	Intergenic		ZNF585A
rs10833529	rs10833529	chr11:21439476	3.38x10^{-06}	Intron	NELL1	
kgp4595579	rs12759252	chr1:63739886	3.38x10^{-06}	Intron	LINC00466	
kgp8452249	rs62105778	chr2:2769920	3.48x10^{-06}	Intergenic		LINC01250
**kgp11866549**	**rs35480289**	chr8:93501729	3.54x10^{-06}	Intergenic		LOC102724710
kgp9260764	rs1454469	chr6:81402538	3.56x10^{-06}	Intergenic		BCKDHB
**rs976145**	**rs976145**	chr6:81407258	3.56x10^{-06}	Intergenic		BCKDHB
rs7593468	rs7593468	chr2:36938368	4.49x10^{-06}	Intron	VIT	
kgp2178065	rs144452299	chr2:48641000	4.79x10^{-06}	Intergenic		PPP1R21
kgp12508031	rs36148756	chr8:15315518	4.94x10^{-06}	Intergenic		TUSC3
kgp19572280	rs17091770	chr14:57066930	5.32x10^{-06}	Intron	TMEM260	
kgp12009830	rs117527115	chr18:68803533	5.44x10^{-06}	Intergenic		LINC01541
rs1327609	rs1327609	chr1:81755444	5.48x10^{-06}	Intergenic		LPHN2
kgp15183992	rs60402019	chr1:17407958	5.68x10^{-06}	Intron	PADI2	
rs28386778	rs28386778	chr17:61964815	6.23x10^{-06}	Intergenic		GH2
kgp8157896	rs150492560	chr2:138282582	6.55x10^{-06}	Intron	THSD7B	
kgp6489287	rs117919802	chr17:68238883	6.57x10^{-06}	Intergenic		KCNJ2
kgp9154489	rs879002	chr7:129196988	6.68x10^{-06}	Intergenic		SMKR1
kgp1193130	rs7821545	chr8:61994173	6.68x10^{-06}	Intergenic		LOC100130298
**rs7144547**	**rs7144547**	chr14:81881735	6.71x10^{-06}	Intron	STON2	
**kgp466155**	**rs142426031**	chr12:81518237	6.74x10^{-06}	Intron	ACSS3	
kgp6972810	rs73180793	chr20:56133517	6.92x10^{-06}	Intergenic		PCK1
kgp12008054	rs6070157	chr20:56137184	6.92x10^{-06}	Exon	PCK1	
rs9513868	rs9513868	chr13:101869226	6.95x10^{-06}	Intron	NALCN	
rs574048	rs574048	chr8:93493264	7.31x10^{-06}	Intergenic		LOC102724710
**kgp2552679**	**rs112374894**	chr14:89008546	7.42x10^{-06}	Intron	PTPN21	
rs10180394	rs10180394	chr2:138293641	7.52x10^{-06}	Intron	THSD7B	
kgp8388119	rs76587784	chr2:138306964	7.52x10^{-06}	Intron	THSD7B	
**kgp4289407**	**rs114123656**	chr2:6747884	7.72x10^{-06}	Intergenic		LINC01246
kgp1447844	rs2854206	chr17:61947203	7.75x10^{-06}	Intergenic		CSH2
kgp10688171	rs144869524	chr2:138175628	7.80x10^{-06}	Intron	THSD7B	
kgp24036278	rs150394950	chr2:138425298	7.80x10^{-06}	Intron	THSD7B	
**rs1560555**	**rs1560555**	chr8:62000239	8.50x10^{-06}	Intergenic		LOC100130298

kgp26381623	rs146855431	chr6:87682167	8.53×10^{-06}	Intron	HTR1E	
kgp2384392	rs116278971	chr2:48678983	8.70×10^{-06}	Intron	PPP1R21	
kgp7759449	rs12547205	chr8:93496111	8.72×10^{-06}	Intergenic		LOC102724710
kgp7136231	rs386727780; rs2000884	chr8:93498680	8.72×10^{-06}	Intergenic		LOC102724710
rs17622419	rs17622419	chr13:101940873	8.92×10^{-06}	Intron	NALCN	
rs1568555	rs1568555	chr3:71193197	9.21×10^{-06}	Intron	FOXP1	
rs12752453	rs12752453	chr1:63738041	9.41×10^{-06}	Intron	LINC00466	
rs12752487	rs12752487	chr1:63738127	9.41×10^{-06}	Intron	LINC00466	
kgp2972657	rs62091597	chr18:53708389	9.50×10^{-06}	Intergenic		LINC01539
kgp11353664	rs13297541	chr9:28087904	9.59×10^{-06}	Intron	LINGO2	
kgp5148773	rs77409462	chr8:68939373	9.68×10^{-06}	Intron	PREX2	
**kgp750736**	**rs13408523**	chr2:36952284	9.75×10^{-06}	Intron	VIT	

[a] SNP ID according to Illumina® HumanOmni5-Quad BeadChip. [b] Positions according to Illumina® HumanOmni5-Quad BeadChip annotations (genome build 37). Boldface values represent SNPs identified as potential leading SNPs in stepwise regression analysis.

Table S2. Associations identified between SNPs and Western dietary pattern.

SNP ID[a]	rs number	Position[b]	*P* value	Localization	Annotated gene	NearestGene
**kgp7827436**	**rs13212846**	chr6:50301100	$4.16x10^{-08}$	Intergenic		DEFB112
kgp9374426	rs116812750	chr1:241484705	$3.40x10^{-07}$	Intron	RGS7	
**kgp9469075**	**rs72736220**	chr4:153093814	$5.35x10^{-07}$	Intergenic		LOC100996286
**kgp1054774**	**rs113152482**	chr10:6278311	$7.88x10^{-07}$	3' near gene region	PFKFB3	
kgp26148321	rs141382233	chr6:157538645	$8.15x10^{-07}$	Intergenic		ARID1B
kgp9033598	rs79041188	chr6:152039889	$2.07x10^{-06}$	Intron	ESR1	
**kgp4441528**	**rs2535974**	chr7:152635944	$2.08x10^{-06}$	Intergenic		ACTR3B
kgp9282379	rs200247	chr6:50401480	$2.54x10^{-06}$	Intergenic		TFAP2D
**rs727628**	**rs727628; rs386791640**	chr16:65327828	$3.68x10^{-06}$	Intron	LINC00922	
kgp27660318	rs140957346	chr12:93249531	$4.05x10^{-06}$	Intron	EEA1	
kgp8552867	rs13218599	chr6:50259937	$4.14x10^{-06}$	Intergenic		DEFB112
rs1348307	rs1348307	chr10:6788713	$4.23x10^{-06}$	Intergenic		LINC00706
rs7911681	rs7911681	chr10:83742695	$4.36x10^{-06}$	Intron	NRG3	
**kgp3463956**	**rs7259811**	chr19:57225019	$5.23x10^{-06}$	Intergenic		ZNF835
**kgp17975556**	**rs115054628**	chr3:4662824	$5.37x10^{-06}$	Intron	ITPR1	
kgp23918889	rs72822576	chr17:14443315	$5.44x10^{-06}$	Intergenic		HS3ST3B1
kgp7404186	rs1559367	chr16:49444459	$5.47x10^{-06}$	Intergenic		C16orf78
kgp7930843	rs2535954	chr7:152631404	$5.71x10^{-06}$	Intergenic		ACTR3B
kgp8978882	rs112040989	chr4:105797467	$6.83x10^{-06}$	Intergenic		LOC101929468
kgp9399667	rs112764838	chr4:105865055	$6.83x10^{-06}$	Intergenic		TET2
kgp6498073	rs112633616	chr12:23238326	$7.95x10^{-06}$	Intergenic		LOC101928441
kgp12198489	rs148295121	chr17:70383353	$8.26x10^{-06}$	Intergenic		LINC00673
kgp29240591	rs148696004	chr4:167164747	$8.38x10^{-06}$	Intergenic		TLL1
**rs9446824**	**rs9446824**	chr6:73737716	$8.58x10^{-06}$	Intron	KCNQ5	
kgp9730403	rs117158239	chr12:23426178	$8.66x10^{-06}$	Intergenic		LOC101928441
**kgp25610618**	**rs140552175**	chr16:88245733	$8.71x10^{-06}$	Intergenic		LOC101928880
kgp12049353	rs10413540	chr19:57226861	$9.60x10^{-06}$	Intergenic		ZNF835

[a] SNP ID according to Illumina® HumanOmni5-Quad BeadChip. [b] Positions according to Illumina® HumanOmni5-Quad BeadChip annotations (genome build 37). Boldface values represent SNPs identified as potential leading SNPs in stepwise regression analysis.

Table S3. Description of gene expression cohort.

Characteristics	**All**	**Men**	**Women**
Number	30	13	17
Age (years)	34.0 ± 9.0	33.5 ± 7.6	34.4 ± 10.2
BMI (kg/m^2)	29.2 ± 3.8	29.1 ± 4.5	29.3 ± 3.3
Waist girth (cm)	90.4 ± 10.3	95.8 ± 10.5	86.2 ± 8.2
<u>Lipid profile</u>			
Total-C (mmol/l)	5.23 ± 0.92	5.36 ± 0.89	5.13 ± 0.96
LDL-C (mmol/l)	3.14 ± 0.94	3.44 ± 0.92	2.91 ± 0.91
HDL-C (mmol/l)	1.40 ± 0.37	1.21 ± 0.37	1.55 ± 0.31
TG (mmol/l)	1.49 ± 0.86	1.54 ± 0.84	1.45 ± 0.90
Total-C / HDL-C	4.04 ± 1.39	4.83 ± 1.56	3.43 ± 0.88
<u>Blood pressure (mm Hg)</u>			
SBP	107.1 ± 10.1	108.1 ± 10.1	106.4 ± 10.3
DBP	70.8 ± 7.7	72.7 ± 7.6	69.3 ± 7.6
Fasting glucose (mmol/l)	4.88 ± 0.54	4.86 ± 0.60	4.90 ± 0.50
Insulin (pmol/)	111.4 ± 156.7	115.7 ± 233.5	77.6 ± 28.4
CRP (mg/L)	4.49 ± 7.08	2.25 ± 1.82	6.08 ± 8.88
<u>Diet scores</u>			
Prudent	0.050 ± 1.054	0.093 ± 1.054	0.016 ± 1.086
Western	0.036 ± 0.820	0.218 ± 0.892	–0.103 ± 0.758

Values presented (means ± SD) are untransformed and unadjusted. Abbreviations: BMI, body mass index; Total-C, total cholesterol; LDL-C; low-density lipoprotein cholesterol; HDL-C, high-density lipoprotein cholesterol; TG, triglycerides; SBP, systolic blood pressure; DBP, diastolic blood pressure; CRP, C-reactive protein.

Table S4. Transcription factors overrepresented in surrounding regions (60 bp) of Prudent dietary pattern-associated SNPs.

Matrix Name[a]	Matrix ID[a]	FDR-corrected *p* value
V$ZNF333_01	M01230	$5.5x10^{-26}$
V$TBP_Q6	M00980	$1.2x10^{-14}$
V$FOXM1_01	M00630	$2.3x10^{-09}$
V$IRF8_Q6	M01665	$8.8x10^{-09}$
V$PAX2_02	M00486	$1.2x10^{-08}$
V$OG2_01	M01162	$2.1x10^{-06}$
V$GR_Q6_01	M00921	$1.1x10^{-05}$
V$HOXA13_01	M01292	$7.1x10^{-05}$
V$RUSH1A_02	M01107	$9.4x10^{-05}$
V$NKX62_Q2	M00489	0.0002
V$CEBPB_01	M00109	0.0002
V$TBP_01	M00471	0.0004
V$CEBP_Q2_01	M00912	0.0004
V$STAT5A_03	M00493	0.0007
V$GATA3_01	M00077	0.0007
V$GATA3_03	M00351	0.001
V$CDXA_01	M00100	0.002
V$FOXP1_01	M00987	0.003
V$XFD1_01	M00267	0.003
V$OCT1_03	M00137	0.003
V$CDXA_02	M00101	0.004
V$HOXA13_02	M01297	0.005
V$SRY_01	M00148	0.005
V$EVI1_05	M00082	0.006
V$IRX5_01	M01472	0.006
V$POU1F1_Q6	M00744	0.006
V$XFD2_01	M00268	0.008
V$IRX2_01	M01405	0.008

V$GFI1_Q6	M01067	0.009
V$PITX2_Q2	M00482	0.009
V$IRXB3_01	M01377	0.01
V$EVI1_06	M00011	0.01
V$XFD3_01	M00269	0.01
V$IPF1_04	M01236	0.01
V$FOX_Q2	M00809	0.01
V$POU6F1_01	M00465	0.01
V$FOXO1_Q5	M01216	0.01
V$FOXO3A_Q1	M01137	0.01
V$RORA2_01	M00157	0.02
V$TEF_Q6	M00672	0.02
V$IRX4_01	M01410	0.02
V$GATA1_02	M00126	0.02
V$FREAC7_01	M00293	0.02
V$OCT1_Q5_01	M00930	0.02
V$NKX32_01	M01181	0.02
V$IRX3_01	M01318	0.03
V$EVI1_03	M00080	0.03
V$OCT_Q6	M00795	0.03
V$MSX1_01	M00394	0.03
V$FOXP3_01	M01599	0.03
V$ATATA_B	M00311	0.03
V$SATB1_01	M01232	0.03
V$HMGIY_Q3	M01010	0.03
V$OCT1_04	M00138	0.03
V$IRX3_02	M01485	0.03
V$HOXA3_01	M00395	0.03
V$CDP_02	M00102	0.03
V$POU3F2_02	M00464	0.03
V$FOXJ2_02	M00423	0.03

V$EN1_01	M00396	0.03
V$EVI1_04	M00081	0.04
V$IPF1_Q6	M01275	0.04
V$EVI1_02	M00079	0.04
V$APOLYA_B	M00310	0.05
V$STAT6_02	M00500	0.05
V$HFH4_01	M00742	0.05

[a] Matrix name and matrix ID according to Transfac database. Overrepresented TF were identified using TRAP multiple sequences.

Table S5. Transcription factors overrepresented in surrounding regions (60 bp) of Western dietary pattern-associated SNPs.

Matrix Name[a]	Matrix ID[a]	FDR-corrected *p* value
V$HMGIY_Q6	M00750	$1.2x10^{-06}$
V$HMGIY_01	M01653	$1.2x10^{-06}$
V$HOXA3_01	M00395	$1.2x10^{-06}$
V$STAT5A_03	M00493	$4.9x10^{-05}$
V$ZNF333_01	M01230	0.0004
V$STAT6_02	M00500	0.002
V$IRF8_Q6	M01665	0.002
V$OG2_01	M01162	0.003
V$IPF1_Q6	M01275	0.01
V$STAT1_03	M00496	0.02
V$HMGIY_Q3	M01010	0.02
V$NKX62_Q2	M00489	0.02
V$TBX15_02	M01264	0.02
V$NFAT1_Q6	M01281	0.04

[a] Matrix name and matrix ID according to Transfac database. Overrepresented TF were identified using TRAP multiple sequences.
